# The Canadian childhood nephrotic syndrome (CHILDNEPH) study: report on mid-study feasibility, recruitment and main measures

**DOI:** 10.1186/s12882-019-1320-3

**Published:** 2019-05-14

**Authors:** Susan M. Samuel, Allison Dart, Guido Filler, Martin Bitzan, Maury Pinsk, Cherry Mammen, Alberto Nettel-Aguirre, Maneka A. Perinpanayagam, Tomoko Takano, Rahul Chanchlani, Michael Zappitelli, other members of the Canadian Childhood Nephrotic Syndrome Project Team, Allison Eddy, Andrew Wade, Anne-Laure Lapeyraque, Catherine Morgan, Ciriaco Piccirillo, Genevieve Benoit, James Tee, Janusz Feber, Pavel Geier, Shannon Scott, Silviu Grisaru, Steve Arora, Daniel Muruve

**Affiliations:** 1 Section of Nephrology, Departments of Pediatrics and Community Health Sciences, Alberta Children’s Hospital Research Institute, O’Brien Institute for Public Health, University of Calgary, 2888 Shaganappi Trail NW, Calgary, Alberta T3B 6A8 Canada; 2 Section of Pediatric Nephrology, Department of Pediatrics and Child Health and Children’s Hospital Research Institute of Manitoba, University of Manitoba, Winnipeg, Manitoba Canada; 3 Division of Pediatric Nephrology, Department of Pediatrics, Medicine, and Pathology & Laboratory Medicine, Western University, London, Ontario Canada; 4 Division of Nephrology, Department of Pediatrics, McGill University, Montreal, Quebec Canada; 5 Section of Pediatric Nephrology, Department of Pediatrics and Child Health, Rady Faculty of Health Sciences, Children’s Hospital Research Institute of Manitoba, University of Manitoba, Winnipeg, Manitoba Canada; 6 Division of Pediatric Nephrology, Department of Pediatrics, University of British Columbia, Vancouver, British Columbia Canada; 7 Departments of Pediatrics and Community Health Sciences, Alberta Children’s Hospital Research Institute, O’Brien Institute for Public Health, University of Calgary, Calgary, Alberta Canada; 8 University of Calgary, Calgary, Alberta Canada; 9 Division of Nephrology, Department of Medicine, McGill University Health Centre, Montreal, Quebec Canada; 10 Division of Nephrology, Department of Pediatrics, McMaster Children Hospital, Hamilton, Ontario Canada; 11 Division of Nephrology, Department of Pediatrics, University of Toronto, Toronto, Ontario Canada

**Keywords:** Nephrotic syndrome, Children, Longitudinal study, Glucocorticoids, Practice variation

## Abstract

**Background:**

To assess reasons for continuing practice variation in the management of childhood nephrotic syndrome despite expert reviews and guidelines, we are conducting a longitudinal cohort study in children with glucocorticoid sensitive nephrotic syndrome.

Objectives of this mid-study report are to describe patient and physician recruitment characteristics, glucocorticoid prescriptions, use of second line agents, biopsy practices, and adherence to study protocol.

**Methods:**

Children with new onset nephrotic syndrome and providers are being recruited from all 12 pediatric nephrology centres across Canada with > 2½ years follow-up. Data collection points of observation are over a minimum 36 months. Details of prescribed glucocorticoids and of all second line agents used during treatment are being collected. All relapses are being recorded with time to urinary remission of proteinuria.

**Results:**

To date, 243 patients (57.1% male) from 12 centres were included. Median number of patients per centre was 29 (range 2–45), and median age of cohort was 7.3 (IQR 4.2) at enrollment. Forty-eight physicians were recruited, median 5 (range 2–8) per site. Median number of relapses per patient year of follow-up was 2.1 (IQR 4). Cumulative dose variability of glucocorticoids prescribed per episode of proteinuria and length of treatment was observed between participating centres.

**Conclusion:**

The Canadian pediatric nephrology community established a longitudinal childhood nephrotic syndrome cohort study that confirms ongoing practice variability. The study will help to evaluate its impact on patient outcomes, and facilitate clinical trial implementation in nephrotic syndrome.

## Background

Childhood nephrotic syndrome is characterized by severe proteinuria and hypoalbuminemia, with edema as the most common presenting symptom [1, 2]. The majority of (> 90%) children with nephrotic syndrome will have minimal change disease and enter remission of proteinuria with glucocorticoid treatment. While the goal of initial therapy is to maximize the proportion of patients with sustained remission, many patients experience relapses (range of 1 to > 20 relapses during childhood) that can lead to significant morbidity [3]. Nephrotic syndrome is associated with increased health system costs which include multi-disciplinary specialist health care team visits, hospital admissions for edema control, infection or vascular thrombosis, and the need for diagnostic kidney biopsies during disease course [4].

The evidence base for treatment of first presentation and relapses of nephrotic syndrome has been synthesized in reviews, local guidelines [5], and in an international clinical practice guideline [6]. There is a lack of consensus regarding best treatment approaches in many aspects of nephrotic syndrome care, including initial treatment, diagnostic approach and treatment of relapses. Our group and others have demonstrated that variation in care is common and that there are centre, physician and patient related factors influencing variation in care [7–9]. However, the impact of existing treatment variation on nephrotic syndrome relapse rates and other outcomes in children remains under studied. Without a careful longitudinal analysis and randomized controlled trials, providers cannot determine whether treatment protocol standardization may lead to improved patient and health care cost outcomes.

We are undertaking a unique, Canada-wide longitudinal cohort study of patients with glucocorticoid sensitive nephrotic syndrome and of the treatment practices of physicians caring for these patients, to address the knowledge gap on the association of nephrotic syndrome treatment variation with relapse rates. The design and methods of this paper have been published previously [10]. The measures used and associations examined in this study are novel, and include evaluating associations between centre-, physician- and patient-level characteristics with glucocorticoid prescriptions, duration of glucocorticoid treatment and relapse rates. Moreover, the feasibility, and recruitment potential were unknown when this national study was launched. Therefore, our objectives are to describe recruitment, patient and physician characteristics, glucocorticoid prescriptions, use of second line agents, biopsy practices, and adherence to study protocol.

## Methods

### Study design and participants

#### Ethics approval and consent to participate

Ethics approval was obtained from the University of Calgary Conjoint Health Research Ethics Board (REB13–0059) in 2013, and subsequently, the study was approved by research ethics boards at all participating sites. Other site ethics numbers are: SickKids Research Ethics Board REB 1000021384; University of Manitoba Bannatyne Campus Research Ethics Board (Health Research Ethics Board – HREB) HS16933 (H2013:420);

University of Saskatchewan Bio-REB # 14–277; University of Alberta Pro00037781;

University of British Columbia Children’s and Women’s Health Research Ethics Board H13–03068; IWK Research Ethics Board 1016972; CHU Sainte-Justine L’approbation éthique 3950; McGill University Health Centre 13–84-PED; Children’s Hospital of Eastern Ontario Research Institute Research Ethics Board 14/188X; Western University Health Science Research Ethics Board 105459; and McMaster University Hamilton Integrated Research Ethics Board15–384. Written parental/guardian consent and patient assent as applicable, were obtained prior to initiating study activities. Written consent was also obtained from physicians before their participation. Funding for the study was provided by the Canadian Institutes of Health Research Project Grant (MOP142271), Kidney Foundation of Canada (140020) and Nephcure Foundation. The funders had no role in the design or conduct of the study, nor in the analysis or interpretation of data.

### Eligibility criteria

All children (age 1–18) who present to the pediatric nephrology clinics with a clinical diagnosis of nephrotic syndrome are eligible for enrollment into this longitudinal cohort study. Twelve major pediatric nephrology centers in Canada are participating in this study and details of these centres have been published previously [10]. In Canada, the majority of patients with nephrotic syndrome are cared for in academic health centres, with a few exceptions in major cities where there are community pediatric nephrologists (1–3 per major site). All pediatric nephrologists who care for children with nephrotic syndrome at participating sites are also eligible for enrollment in order to evaluate physician characteristics specific to each prescription.

Inclusion and exclusion criteria for patients are shown in Table 1. Patients may be enrolled at first presentation of nephrotic syndrome or at their first or second relapse. Patients < 1 year of age are excluded due to higher prevalence of genetic cause of nephrotic syndrome and probability of glucocorticoid resistance. We also exclude patients with secondary nephrotic syndrome due to other diseases such as IgA nephropathy and systemic lupus erythematosus.

**Table 1:** Inclusion and exclusion criteria for study

		First Presentation	First Relapse	Second Relapse
Inclusion Criteria	*Age*	1 to ≤17.5 years	1 to ≤17.5 years	1 to ≤17.5 years
	*Proteinuria*	> 3+ on dipstick; > 3 g/L on urinalysis; urine protein to creatinine ratio > 200 mg/mmol, for 3 consecutive days	> 3+ on dipstick; > 3 g/L on urinalysis; urine protein to creatinine ratio > 200 mg/mmol, for 3 consecutive days	> 3+ on dipstick; > 3 g/L on urinalysis; urine protein to creatinine ratio > 200 mg/mmol, for 3 consecutive days
	*Serum Albumin*	< 25 g/L	< 25 g/L	< 25 g/L
	*Exposure to Glucocorticoids*	No prior exposure to glucocorticoids	Could have prior exposure to glucocorticoids (at First Presentation)	Could have prior exposure to glucocorticoids (at First and Second Presentation)
	*Glucocorticoid sparing agents used*	None	None	None
Exclusion Criteria	A primary disease associated with nephrotic syndrome (e.g. lupus, malignancy)			
	Serum C3 concentration low; suggesting alternative cause of nephrotic syndrome			
	Patients ultimately shown to be glucocorticoid resistant will be excluded from the final analysis (but will continue to be followed)			

### Enrollment period

Enrollment began at the first participating site in August 2013, and all remaining sites started recruitment in the following 12 months. Enrollment will continue until December 2019, for a target recruitment of 400 patients. The observation period will be extended for an additional 6 months after the enrollment period (until June 2020, to ensure a minimum 6 months follow-up for all patients by the end of study).

### Data collection time points

**Fig. 1:**
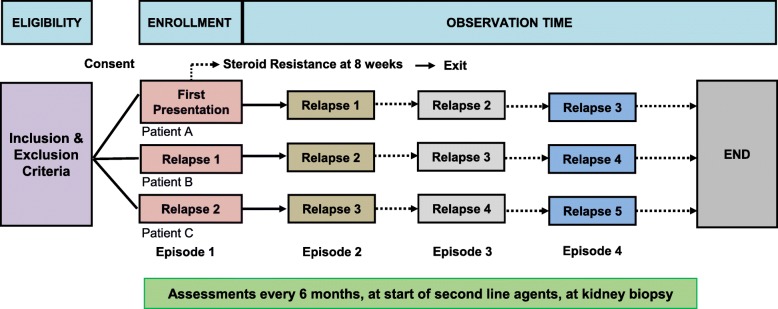
Data collection time points during observation

Study data are being collected at the following time points in the patients’ disease course: 1) at study entry (first presentation, first relapse or second relapse); 2) at the beginning of all relapses occurring during the observation period [defined as either proteinuria > 3+ on urine dipstick, > 3 g/L by quantitative urine chemistry, or urine protein to creatinine ratio > 200 mg/mmol, for 3 consecutive days or start of full dose glucocorticoids (60 mg/m^2^/day or 2 mg/kg/day)] [6]; 3) at the end of first presentation and end of all subsequent relapses [defined as both remission of proteinuria – negative or < 0.3 g/L [< 1+] protein on dipstick for 3 consecutive days, and off glucocorticoids] [6]; 4) study visits every 6 months during remission; 5) at start of all glucocorticoid sparing agents; 6) at time of all kidney biopsies; and 7) study end (defined by end of observation, discharge from clinic or exclusion due to identification of an underlying diagnosis as a cause of nephrotic syndrome). For patients recruited at first or second relapse, the data from first presentation and/or first relapse are being collected retrospectively. The data collection timelines are outlined in Fig. 1.

### Definition of a study “episode” and data collected during episodes

**Fig. 2:**
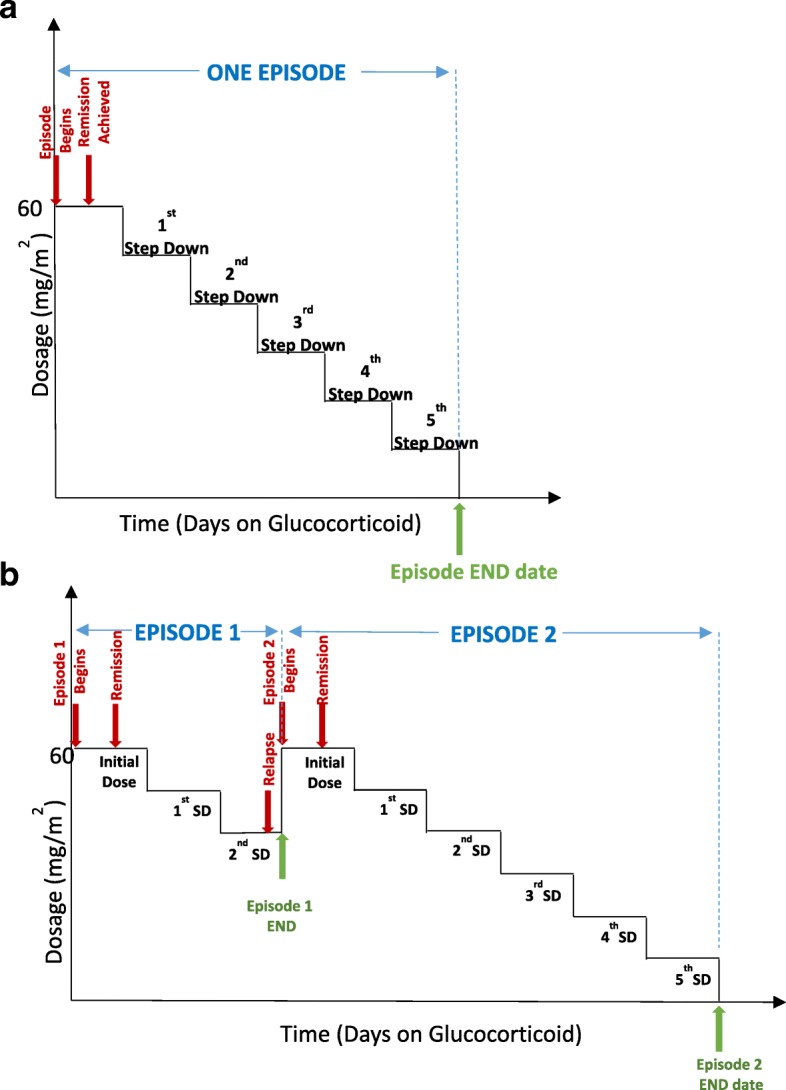
Defining an episode, illustrating sample scenarios. An episode is defined as the time from start of full dose glucocorticoid therapy (60 mg/m^2^ or 2 mg/kg) to cessation of glucocorticoids (**a**) or re-start of full dose glucocorticoids as in glucocorticoid dependent patients who relapse while tapering glucocorticoids. (**b**) Note that the glucocorticoid tapering mode may vary between prescribers. Abbreviation: SD – step down

An ‘episode’ refers to glucocorticoid treatment schedule used for the first presentation of nephrotic syndrome and all relapses. Therefore, an episode will begin at the time of start of full dose glucocorticoid therapy (60 mg/m^2^/day or 2 mg/kg/day) and will continue until cessation of glucocorticoids or re-start of full dose glucocorticoids such as in patients who relapse while tapering glucocorticoids during an episode. Figure 2 illustrates definition of episodes in the study. Each prescription protocol is recorded by entering the total dose prescribed for specific dates, with all changes in dose for the taper. For each episode, we are recording urine dipstick results (in SI units) at the start of treatment with glucocorticoids, and relapses are defined as above using standard definitions. Data regarding urine remission dates are also being recorded. For both relapse and remission, home or point-of-care dipstick urine protein and/or urine dipstick results from laboratory testing, or protein to creatinine results are acceptable in the study protocol. Patient height and weight are recorded with each prescription to allow for calculation of body surface area or mg/kg dosing.

Prescriptions are linked to the physician who prescribed it if he/she consented to be part of the study, and all physicians are grouped by the participating center. For physicians who either declined consent or were filling in temporary positions (locums), we did not collect or record identifying information and coded their prescriptions as ‘unknown’ physician in the study with no known demographics.

Therefore, the study dataset is hierarchical in that patients are linked to the physician who prescribed glucocorticoids, and physicians and patients are grouped by site.

Health data are collected during clinic visits to nephrologists (every six months after entry into study), including, height, weight, interim medical history (changes in health status, revision of diagnosis, new diagnoses), and urine protein-to-creatinine or albumin-to-creatinine ratios (only in one centre) during remission. Dose and duration of treatment (start and end dates) with all second line agents (e.g. pulse intravenous methylprednisolone, cyclophosphamide, tacrolimus, cyclosporine, mycophenolate, rituximab, levamisole) are recorded in the data.

### Analysis

Due to the hierarchical nature of our dataset, we analysed the data at 3 levels – patient, physician, and site. We used means, medians and proportions to describe the patient, physician cohort and site characteristics according to key demographics. The patient recruitment over calendar years will be reported.

Episodes as defined in this study includes both first presentation and relapses. Relapses were defined as all episodes recorded apart from first presentation episodes. Median number of relapses per person year of observation for the cohort was calculated.

We determined glucocorticoid exposure for the entire cohort and for patients grouped by site. For the cohort, we determined the median glucocorticoid exposure for first presentation and relapses (dose and duration of therapy). For the purpose of the study, prednisone and prednisolone were used equivalently. We reported variation in cumulative prednisone or prednisolone prescribed for episodes and length of episodes by site in mg/m^2^, when all patients within a site were grouped. Maximum and minimum median values between sites were determined, and variation within site was depicted using box plots. Finally, we reported summary statistics of the use of second line agents and biopsy practices observed in the cohort, as well as measures of adherence to study protocol. We did not impute values for missing data. We analysed all data until the last active follow-up date (defined by a completed episode or semi-annual visit).

## Results

### Patient cohort descriptors

**Fig. 3:**
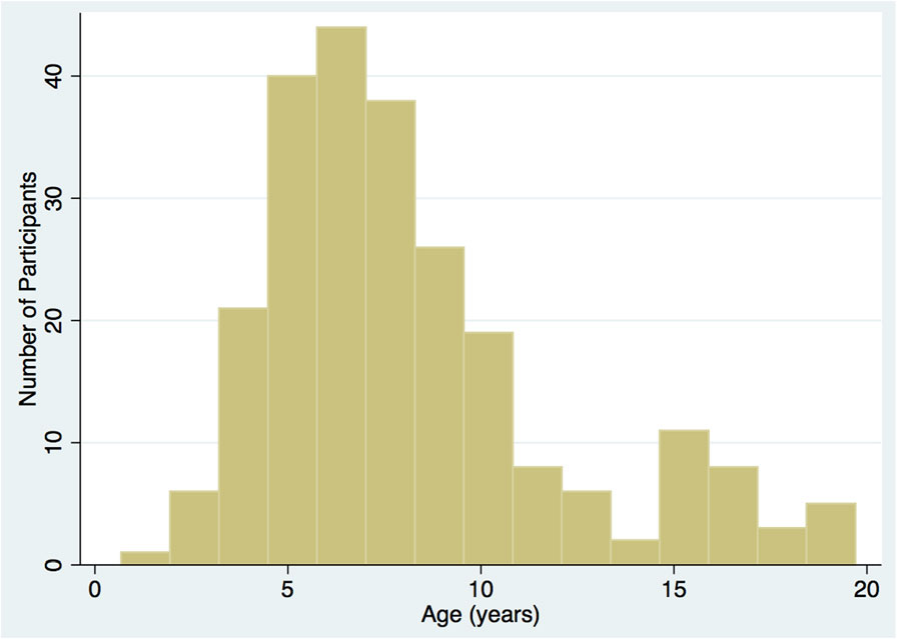
Distribution of age at enrollment into the study

Between August 2013 and August 2017, a total of 269 patients were screened and 262 were found to be eligible. Of these, 243 (92.8%) were enrolled into the cohort. For the remainder, 10 declined consent, 3 did not consent due to language barrier, and 6 were not consented for other reasons. Out of 243 enrolled, 215 (88.5%) were enrolled at first presentation, 18 (7.4%) at first relapse, and 10 (4.1%) at second relapse. The median age was 7.3 (IQR 4.2) years at enrollment; the variability in age at enrollment is shown in Fig. 3. There were 140 (57.6%) boys and 103 (42.4%) girls. The median patient years of follow-up in the cohort was 1.0 (IQR 1.6) years. We are enrolling primarily patients at first presentation currently (78% new presentations in 2014 versus 97% new presentations in 2017). The study protocol mandates that all eligible patients at each site are screened for enrollment. It is possible that in some sites that all patients who presented were not screened due to lack of a study coordinator for certain periods of time. The number of missed patients at each site is expected to be low due to the rare incidence of disease in general. Of note, the largest site SickKids Hospital in Toronto agreed to enroll 30 patients due to feasibility and funding issues, based on an *a prori* agreement with study team.

**Fig. 4:**
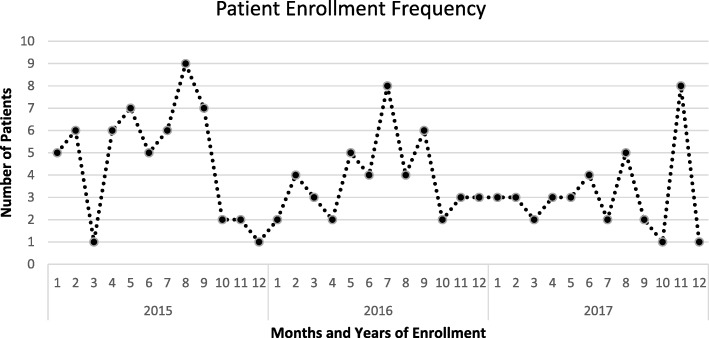
Seasonal variation in enrollment and all episodes recorded

The average recruitment rate over the calendar years of September 2013 to December 2017 was 3.9 cases/month. Monthly variation in enrollment and episodes recorded were noted as seen in Fig. 4.

### Physician cohort descriptors

There were a total of 78 physicians registered in the Canadian Association of Pediatric Nephrologists list at the time of study initiation, of whom 10 were inactive due to retirement and lack of a clinical practice. Among the 68 remaining physicians, 50 were deemed eligible and approached for the study as they were identified as being involved in providing care to patients with nephrotic syndrome within an academic health centre setting. Ineligible physicians were pediatric nephrologists who are practicing in the community, not engaged in clinical practice (academic positions without clinical care), or do not provide care to nephrotic syndrome patients through ambulatory care. Of those eligible, 48 physicians consented to be enrolled.

The physician cohort included 24 males and 24 females; of these 96% work full-time, 48% were between 40 and 50 years of age and 75% trained in Canada only. Individual physicians wrote an mean of 16.6 (standard deviation 13.8, range 1–54) prescriptions. There were only 4 episodes recorded in the data prescribed by an ‘unknown’ physician.

### Site cohort descriptors

The median recruited number of patients by site was 29 (IQR 28, range 2–45) and the median recruited number of physicians by site was 5 (range 2–8) respectively. There were 5 sites in Western Canada (4 Provinces), 4 sites in Ontario, and 3 sites in Quebec and Eastern Canada (3 Provinces). The enrollment numbers roughly represent the size of the centre, except for the largest site (SickKids Hospital, Toronto) which contributed 30 patients based on a priori recruitment target. Sites with coordinators (6 in total) have a consistent record of enrolling patients throughout the period of the study. The remaining 6 have periods of lack of enrollment and in many instances they are able to back fill recruitment if the patient still fit criteria for enrollment once a coordinator is available. In general, the enrollment numbers reflect the volume of patients seen at that site.

Among these sites, 7 reported that they were using protocol based treatments for nephrotic syndrome. The remainder reported that each physician used a prescription that was unique to that physician’s style.

### Patient relapse data

The total number of episodes (which includes both first presentation and relapses) observed was 816 in 243 patients. The total number of observed relapses was 609 in 243 patients. Overall, median number of relapses per patient year of follow-up was 2.1 (IQR 0–4). Of 243 patients, 15 (6.4%) were deemed to have glucocorticoid resistance during observation and were excluded from relapse frequency and glucocorticoid exposure calculations.

After assuming a minimum follow-up of one year for all patients in this mid-study analysis (to minimize over-inflation of reported rates due to short lengths of follow-up), there were 90 (41.3%) patients who experienced ≤1 relapse observed per patient-year of follow -up (group 1), 100 (45.9%) patients who experienced 1.1 to 3.9 relapses per patient-year of follow-up (group 2), and 28 (12.8%) patients who experienced ≥4 relapses per patient year of follow-up (group 3).

### Glucocorticoid exposure data

Among the 816 episodes captured, 731 (90%) had complete glucocorticoid prescription data entered and were used for the glucocorticoid exposure analyses (185 first presentation and 546 relapses). The median cumulative glucocorticoid exposure for first presentation episode was 3138 (IQR 1333) mg/m^2^, and relapse episodes were 1259 (IQR 756) mg/m^2^ in cohort. The median length of treatment was 96 days (IQR 39) for first presentation episode and 51 days (IQR 36) days for relapse episodes in the cohort.

**Fig. 5:**
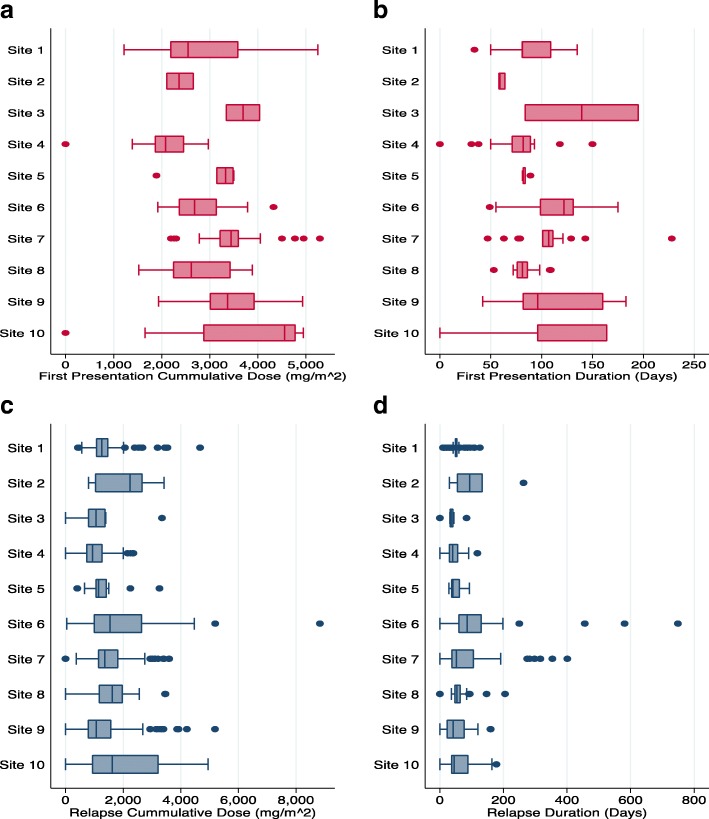
Site specific differences of cumulative dose of glucocorticoid given (**a**) and the length of treatment (**b**) at first presentation. Further illustrated are the site-specific differences of cumulative dose of glucocorticoid given (**c**) and length of treatment (**d**) at relapse. Data from 2 sites were not shown due to low enrollment at the time of this report. Note: ^I^ Sites 1, 3,5,6,7,9,10 used protocol based treatments for nephrotic syndrome while Sites 2, 4, 8 did not. ^II^ Number of patients for sites 1–10 in first presentation graphs were 45, 3, 2, 37, 8, 29, 30, 22, 36 and 29 respectively. ^III^ Number of patients for sites 1–10 in relapse graphs were 32, 2, 1, 22, 6, 20, 22, 17, 24 and 18 respectively

Figure 5 shows that the cumulative glucocorticoid dose (in mg/m^2^) per episode (either first presentation or first relapse), varied by site when patients within a site are grouped. The overall correlations between the cumulative glucocorticoid dose per episode for patients within a site were 0.32 [95% CI 0.14–0.57] and 0.09 [95% CI 0.028–0.26] for first presentation and relapse respectively. The median absolute deviation (MAD) for the cumulative glucocorticoid dose within sites for first presentation episode ranged from 153.4 mg/m^2^ to 820.9 mg/m^2^ and for relapses ranged from 98.3 mg/m^2^ to 805.9 mg/m^2^.

### Second line agents and biopsy practices

Of 243 patients enrolled, 96 (39.5%) patients started treatment with a second line drug at a median time of 10.8 (IQR 13.6) months from first episode recorded in the dataset (either first presentation, first or second relapse). The most common second line agent used was tacrolimus (49, 33.3%), followed by oral cyclophosphamide (35, 23.8%), mycophenolate mofetil (26, 17.7%) and rituximab (22, 14.9%). Among those who received second line agent, 19 patients received more than one drug (1 patient received 4, 2 patients received 3, and 16 patients received 2 different second line agents). Rituximab was given at a median time of 15.8 (IQR 23.5) months from first episode observed.

Biopsies were performed in 56 (23%) children, and two children had two biopsies. Among the 58 biopsies, most common reasons for biopsy include initial diagnosis (13; 22.4%), glucocorticoid resistance (11; 18.9%), and glucocorticoid dependence (9; 15.5%). Minimal change disease (36; 62%) was the most common histopathologic diagnosis, followed by focal segmental glomerulosclerosis in 7 (12%) children. Other histological diagnoses were acute tubular necrosis, membranous nephropathy, IgA nephropathy, and C1q nephropathy.

### Study protocol adherence

At least one semi-annual visit (routine health data collected during clinic visits to nephrologists every six months after entry into study) was documented in 208 of 243 (85.6%) enrolled patients, and all of them had at least one visit not recorded (missing). Of the 208 patients, 11 (5.3%) had < 25% visits completed, 60 (28.8%) had 25 to 75% visits completed and 137 (65.9%) had > 75% visits completed. Of the semi-annual visits data forms that were completed, weight and height data were recorded in 97 and 98% of patients respectively. Compliance with completion of data were similar between sites with and without dedicated research coordinators (6 of 12 sites) for the study (data not shown).

## Discussion

The Canadian Childhood Nephrotic Syndrome Project has been successful in recruiting patients from major pediatric nephrology centres across Canada. We are more than half-way to our recruitment goal of 400 patients. We have sufficient data to understand pattern of glucocorticoid prescriptions, relapse rates, second line agent use, and biopsy practices. Protocol adherence is variable. Site and physician based variation was notable in glucocorticoid exposure; however, full results regarding how variation affects relapses will be available in the year 2020.

We designed this study in the context of known widespread practice variation within Canada and other jurisdictions among providers and centres in the management of nephrotic syndrome [8]. Such variability should also be taken into consideration when designing protocols to minimize glucocorticoid exposure, which is an identified patient priority [11]. With the new data from this study, we can determine the strength of associations between centre-, physician-, and patient-level characteristics with glucocorticoid prescriptions, and relapse rates. The final results, combined with recent clinical trial data which shows that longer duration of steroid treatment and/or higher dose (8 weeks of treatment) does not reduce relapse rate [12–14], will help us draft consensus protocols for the Canadian pediatric nephrology community, which will aim to minimize glucocorticoid toxicity and maximizing efficacy in averting relapses.

Despite an overall high consent rate (> 90%) from eligible patients, the current recruitment rate would suggest that we will not reach our target enrollment by end of the study (December 2019). We have seen significant variability in incidence of disease over time with spikes or lulls in incidence of disease consistently across enrolling centers (e.g. when incidence is low, it tends to be low at all centres). Therefore, challenges in attaining the target recruitment may be due to the sporadic incidence of nephrotic syndrome. Further, variability and turn over in research staff at each site has been identified as a challenge for recruitment throughout the time period of the study. For sites with coordinators, we are confident that all potentially eligible patients have been approached. There are gaps in enrollment at sites where coordinators were absent for a period of time. To overcome these challenges, we are planning to extend recruitment period as necessary and apply for new funding to keep the study open.

Compliance with data entry is variable across centres. Availability of research staff to enter data and follow patient’s clinical course in real time is important, but does not lead to complete data entry at all sites with coordinators. We also identified challenges in completing records for semi-annual visits (only 66% with > 75% of expected visits completed) and entering of full glucocorticoid taper data (only 90% complete) at the end of treatment. We have not provided specific funding to conduct ‘research visits’ and are relying on routine care visits to occur in order for us to capture semi-annual data – this poses a barrier to collection of complete data. Measures to improve data collection completeness and protocol adherence in the future includes creation of quarterly data quality reports and real time data entry monitoring by central study staff, and systematic provision of feedback to sites to complete missing data.

One of the most important characteristics of this study, and its strength, is its focus on patients with glucocorticoid sensitive disease early in their disease course. Most nephrotic syndrome registries focus on glucocorticoid resistant patients and those who have had a kidney biopsy [15, 16]. These are a minority among the larger group of patients with nephrotic syndrome in childhood, as most (80%) are typically not biopsied at presentation [1]. Therefore, many registries contain a biased sample of patients seen in pediatric nephrology clinics. With successful recruitment, we have the opportunity to collect a heterogeneous sample of patients, early in the course of disease. We acknowledge that some patients may be seen by community-based pediatricians (true estimate unknown, and difficult to ascertain) in large urban centers (Vancouver, Toronto) as well as in remote areas (particularly smaller provinces in Atlantic Canada with no pediatric nephrologist). Despite these exceptions, there is a well-established culture of referral to pediatric nephrology of children with nephrotic syndrome in almost all major pediatric health centres in Canada.

Our focus in this paper is to report on mid-study feasibility and not to report on results. However, some results are notable even at this stage. We are observing a consistent relapse rate of approximately 2 per patient year of follow-up. There is wide variation in cumulative dose of glucocorticoid prescribed between and within sites for initial presentation. The variation is less marked for relapses. This is an important observation that needs to be considered in light of recent trial evidence which shows that longer glucocorticoid duration or higher dose does not reduce relapse rate [13, 14, 17]. We acknowledge that practice is also changing based on emerging evidence as we continue the study, and we will be capturing this data. However, this observational data, taken together with recent trial evidence, point to the need to address the optimal dose and duration for initial presentation and relapse using both evidence and consensus based approaches.

Our study has a number of additional strengths. Recruitment of physicians and linkage to patient data, collection of detailed glucocorticoid prescriptions, and enumeration of all relapses with the outcome of treatments are a strength. In addition, the study infrastructure will allow us the opportunity to collect blood and urine samples, in particular relapse remission paired samples in order to conduct translational studies. The combination of carefully collected clinical data, combined with potential for biomarker data to be generated from blood and urine samples will help us study pathogenesis of disease and develop targeted treatments in order to achieve transformational change in the management of nephrotic syndrome.

The infrastructure built for this study was specifically designed to conduct registry based clinical trials [18]. Once registered into this study, randomization of patients for future clinical trials, and refining protocols for treatment of first presentation and relapses becomes easily possible.

There are also several limitations to consider. We are limited by the observational design of this study. There may have been some referral bias, especially in larger urban centres where community physicians are seeing patients with nephrotic syndrome. These patients are not included in the study due to feasibility of having study coordinators on site at community practices, and therefore some analysis such as seasonal variation in incidence may have a degree of error due to under-reporting. We do not attempt to classify patients in terms of relapse frequency as per convention for nephrotic syndrome (frequently relapsing, steroid dependent), as we are describing the phenotype of the patients in an ongoing longitudinal study. The follow-up of patients in the cohort has been sporadic in some centres due to lack of access to research staff. We are not measuring patient adherence to treatment. It is acknowledged that adherence to treatment may be a major contributor to relapse risk, and since our data do not contain this information adherence will be an unmeasured confounder. We are also not following patients after transfer to adult care, due to funding limitations.

## Conclusion

A longitudinal cohort study regarding idiopathic childhood nephrotic syndrome has been successfully launched in Canada. Full results are expected in 2020. The infrastructure of this study will be a key enabler for registry based clinical trials and will spur innovative translational studies regarding pathogenesis and treatment of nephrotic syndrome.

## Abbreviations

IQR: Interquartile range
SD: Standard deviation
SI units: Standard international units
